# Editorial Comment to Combination therapy with radiation and hyperthermia‐induced clinical complete response of small cell carcinoma of prostate

**DOI:** 10.1002/iju5.12417

**Published:** 2022-01-18

**Authors:** Sunao Shoji

**Affiliations:** ^1^ Department of Urology Tokai University School of Medicine Isehara Kanagawa Japan

Small cell carcinoma of the prostate (SCCP) is usually treated with chemotherapy, such as cisplatin/etoposide, carboplatin/etoposide, docetaxel/carboplatin, and participation in clinical trials. However, it would be difficult to introduce chemotherapy in elderly patients and patients who have other diseases that affect chemotherapy, such as renal dysfunction and heart failure. The authors reported their experience on whole‐pelvic radiation therapy with an additional dose administered to the prostate and concurrent 8‐MHz radiofrequency capacitive regional hyperthermia for sensitizing the effect of radiation therapy for an 87‐year‐old man with SCCP.^1^

As other modalities of hyperthermia for prostate cancer, high‐intensity focused ultrasound (HIFU) has been used for whole‐gland^2^ and focal therapies.^3^ HIFU is an extracorporeal ablative technology that delivers ultrasonic energy to pinpoint the foci only millimeters wide, and only minor temperature changes from 70 to 98.6°C are observed in the focal zone.^3^, ^4^ The 8‐MHz radiofrequency capacitive regional hyperthermia warms the wide part sandwiched between electrodes to 42°C. Due to the present case having bladder invasion and right obturator lymph node metastasis of SCCP, radiofrequency hyperthermia was appropriately used due to the wide heat effect around the prostate. The safety of the surrounding large abdominal vessels during the radiofrequency hyperthermia^5^ encouraged the treatment for the lymph node near the large blood vessels.

As a result, the authors encountered an effective case of SCCP treated with a combination of radiotherapy and hyperthermia therapy. For patients who have difficulty in receiving chemotherapy, the present treatment has the possibility to be the novel strategy for SCCP. I would like to pay tribute to the authors’ attitude toward patients and their challenging spirit.

## Conflict of interest

The author declares no conflict of interest.
